# Neuroprotective Effects of Deproteinized Calf Serum in Ischemic Stroke

**DOI:** 10.3389/fneur.2021.636494

**Published:** 2021-09-07

**Authors:** Weiwei Li, Anchen Guo, Ming Sun, Jiachuan Wang, Qun Wang

**Affiliations:** ^1^Department of Neurology, Beijing Tiantan Hospital, Capital Medical University, Beijing, China; ^2^China National Clinical Research Center for Neurological Diseases, Beijing, China; ^3^Department of Surgery, University of Cincinnati, Cincinnati, OH, United States; ^4^Beijing Key Laboratory of Translational Medicine for Cerebrovascular Disease, Beijing, China; ^5^Beijing Institute of Brain Disorders, Collaborative Innovation Center for Brain Disorders, Capital Medical University, Beijing, China; ^6^Department of Neuropharmacology, Beijing Neurosurgical Institute, Beijing, China; ^7^Department of Pathology, Shenzhen Traditional Chinese Medicine Hospital, The Fourth Clinical Medical College of Guangzhou University of Chinese Medicine, Shenzhen, China

**Keywords:** cerebral ischemia, deproteinized calf serum, Actovegin, apoptosis, reactive oxygen species

## Abstract

Deproteinized calf serum (DCS) may have neuroprotective effects after ischemic stroke. The aim of this study is to investigate whether and how the DCS inhibits neuronal injury following cerebral ischemia. Rats were subjected to 2 h transient middle cerebral artery occlusion (MCAO). One dose of 0.125 mg/gbw DCS was given immediately after reperfusion. Neurological deficit and infarct volume at 24 h post-MCAO in DCS-treated rats were lower than those in vehicle-treated rats (*p* < 0.0005). In cultured neurons model, cell viability was decreased, and apoptosis was increased by oxygen-glucose deprivation/reperfusion (OGD/R) (*p* < 0.0005). These effects of OGD/R were attenuated by 0.4 μg/μl DCS (*p* < 0.05) that were validated by CCK8 cell viability assay, phycoerythrin–Annexin V Apoptosis Detection assay, and TUNEL assay. Furthermore, the increase of intracellular ROS level in cultured neurons was suppressed by DCS (*p* < 0.05). Compared with cells subjected to OGD/R, the expression level of Bax protein decreased, and bcl-2 protein increased after DSC treatment (*p* < 0.05). Overall, the neuroprotective effects of DCS following cerebral ischemia may in part be due to decreased ROS production and inhibition of apoptosis.

## Introduction

Stroke is an acute disorder caused by abnormal blood supply to the brain and a major cause of morbidity and mortality worldwide [(1), p. 161–176]. Stroke is the fifth leading cause of death and main cause of disability in adults in the USA [(2), p. 165260]. Recent estimates suggest that nearly 795,000 individuals experience a stroke every year in the USA, corresponding to one victim every 40 s [(2), p. 165260]. Ischemic stroke accounts for 60–80% of all cases, and hemorrhagic stroke accounts for the remainder [(3), p. 7–32]. The main aim of therapy for acute ischemic stroke is to achieve rapid revascularization and thus restoration of blood flow [(4), p. 967–973, (5), p. 1066–1072]. Thrombolysis with recombinant tissue-type plasminogen activator (tPA) within a few hours of symptom onset is recognized as a potentially effective treatment for acute ischemic stroke and widely used in the clinical setting [(6), p. CD000213]. Surgical treatments have also been developed, including retrievable self-expanding stents for thrombectomy of acute proximal intracranial artery occlusions [(7), p. 12–20]. However, despite costing more than 70 billion dollars per year in the USA alone, the available treatments are limited in scope and predominantly supportive in nature [(2), p. 165260].

Ischemic stroke results from the blockade of an artery to the brain from *in situ* thrombosis or an embolus from another artery or the heart. Due to the brain's limited capacity to store glucose and utilize anaerobic metabolism, the blood flow deficit leads to rapid neurodegeneration and an expanding infarct core surrounded by potentially salvageable penumbral tissue that is the primary target for treatment strategies [(8), p. 975–980]. The development of novel therapies requires an understanding of the processes underlying the brain injury that follows stroke. These mechanisms include excitotoxicity, acidotoxicity, ionic imbalance, oxidative stress, nitrative stress, inflammation, and apoptosis [(9), p. 297–309, (10), p. 1167–1186]. Neuronal apoptosis, a major mechanism of cell death induced by cerebral ischemia/reperfusion (I/R) injury and the balance between the expressions of antiapoptotic Bcl-2 protein and proapoptotic Bax protein, is critical for regulating apoptotic cell death [(11), p. 1334–1340]. Bcl-2 exerts a survival function in response to a wide range of apoptotic stimuli through inhibition of mitochondrial cytochrome c release. Bax is a key component for apoptosis induced by mitochondrial stress. Upon stimulation of the apoptotic pathway, Bax forms oligomers and translocates from the cytosol to the mitochondrial membrane to interact with pore proteins in the mitochondrial membrane and increases the membrane's permeability. This leads to the release of cytochrome c from the mitochondria, activation of caspase-9, and initiation of the caspase-dependent apoptotic pathway.

Oxidative stress and mitochondrial dysfunction are widely recognized as making an important contribution to neuronal apoptosis following I/R injury. Dysfunction of the mitochondrial respiratory chain during ischemia leads to the generation of reactive oxygen species (ROS) [(12), p. 712–718]. Numerous *in vivo* and *in vitro* studies have provided evidence implicating important roles for ROS and mitochondrial-dependent apoptosis in the death of neuronal tissue following I/R injury [(13), p. 98–106, (14), p. 1491–1499].

Deproteinized calf serum (DCS, also known as Actovegin, AODEJIN® Avanc Pharmaceutical Co., Ltd., Jinzhou, China), a deproteinized ultrafiltrate of calf blood composed of more than 200 biological substances, has been used in clinical practice for a variety of indications including ischemic stroke and brain injury, peripheral arterial and venous perfusion disorders, diabetic polyneuropathy, and skin trauma [(15), p. 80–88]. DCS has been reported to have multiple metabolic effects, including improved oxygen utilization and uptake, enhanced cellular energy metabolism, and increased glucose uptake and oxidation [(16), p. 266–274]. These effects may contribute to the beneficial effects of DCS in patients with diabetic polyneuropathy [(17), p. 1181–1187]. Therefore, it is possible that DCS may exert a neuroprotective effect by enhancing energy metabolism in the brain after ischemia or injury. DCS has been reported to reduce focal neurologic deficits in patients with ischemic stroke, enhance cognitive function in patients with ischemic stroke [(18), p. 873–875] or vascular mild cognitive impairment, improve the functional rehabilitation of patients after ischemic and hemorrhagic stroke, correct immunometabolic disturbances in patients with chronic cerebral ischemia, and facilitate recovery after brain injury. In addition, DCS was found to enhance cell survival in the hippocampal CA1 region and improve spatial learning and memory in rats subjected to transient forebrain ischemia [(19), p. 1623–1630]. DCS has also been reported to reduce oxidative stress, inhibit apoptosis, and maintain excitatory synapses (in a concentration-dependent manner) in cultured primary rat neurons challenged with amyloid peptide Aβ [(16), p. 266–274]. DCS was also observed to attenuate H_2_O_2_-induced apoptosis of human neuroblastoma cells [(20), p. 215–217].

We hypothesized that the neuroprotective effects of DCS may be mediated, at least in part, by reduced generation of ROS. Therefore, the present study utilized a rat model of ischemic stroke and cultured neuronal cells to investigate whether DCS inhibited neuronal injury following ischemia by reducing ROS and suppressing apoptosis.

## Materials and Methods

### Animals

Thirty-six Sprague–Dawley male rats (weighing 320–340 g) and 12 E16–18 pregnant Sprague–Dawley rats were obtained from the Vital River Laboratory Animal Technology Co., Ltd., Beijing, China. The animals were housed at 20–25°C and 50 ± 5% humidity with a 12:12-h light/dark cycle and were given *ad libitum* access to food and water. All animal experiments complied with ethical requirements and were approved by the ethics committee of the Beijing Tiantan Hospital of Capital Medical University.

### Rat Model of Ischemic Stroke

Transient middle cerebral artery occlusion (MCAO) was used as the rat model of ischemic stroke, and the procedure was based on a modification of previously described methods [(21), p. 84–91]. Briefly, rats were anesthetized with 5% isoflurane delivered in air for 2 min. Anesthesia was maintained with 2–3% isoflurane delivered in air at 0.5 L/min during surgery. Body temperature was continuously monitored with a rectal probe and maintained at 36.5–37.0°C using a temperature-controlled blanket. Then, 0.1 mg/kg buprenorphine was given pre-emptive to reduce the animal's pain, suffering, and distress. Humane endpoints were met if the animal's excessive blood loss or drop in body temperature or more than 20% of its body weight lost within 8 h post-operation. The right common carotid artery (CCA), external carotid artery (ECA), internal carotid artery (ICA), and vagus nerve were exposed and isolated carefully under a dissecting microscope. Flows through the CCA and ICA were temporarily stopped using a slipknot, and the distal end of the ECA was electrocauterized and resected. Then, a small hole was cut on the proximal end of the ECA, and a 3-0 silicon rubber-coated monofilament was inserted *via* the ECA into the lumen of the ICA. After loosening of the slipknot on the ICA, the filament was advanced 18–20 mm beyond the carotid bifurcation to the middle cerebral artery (MCA). After 2 h of occlusion, the filament was withdrawn, the hole in the ECA was sealed by electrocauterization, and the slipknot on the CCA was loosened to allow reperfusion.

Sham animals were anesthetized with isoflurane, and the CCA, ECA, lCA, and vagus nerve were exposed and isolated carefully, and then the incision was closed. Model animals were randomly divided into two groups and kept in different cages with clear labels: Model and Model + DCS. For rats in the Model + DCS group, one dose of 0.125 mg/gbw DCS (Avanc Pharmaceutical Co., Ltd., Jinzhou, China) was injected intraperitoneally immediately after reperfusion, and the brains were collected after 24 h [(22), p. 105–110, (23), p. 256–266, (24), p. 1638-018-20095–9]. There were 12 animals per group, and the animals were monitored every 8 h with food and water accessible. Animals that do not show clinical signs of focal cerebral ischemia (i.e., hemiparesis or circling), indicating insufficient vessel occlusion, were excluded. Rats in the Model group only received an injection of the same amount of saline (vehicle).

### Assessment of Neurological Function in Rats Subjected to MCAO

Neurological examinations were performed at 24 h after reperfusion by two observers who were blinded to the animal grouping. The assessment of neurological dysfunction (neurological deficit score) was made using the modified Bederson's method [(25), p. 472–476].

### Measurement of Infarct Volume Using Triphenyltetrazolium Chloride

Each rat was sacrificed after the neurological examination had been completed, and the brains of 18 animals (6 per group) were removed and cut into 2-mm thick coronal sections (the remaining animals were used for Western blot testing). The brain sections were incubated in a solution of 1% triphenyltetrazolium chloride (TTC) in normal saline for 20 min at 37°C. The slices were photographed using a Leica microscope (Leica, USA), and the infarct size was determined.

### Culture of Primary Rat Neurons

Primary neurons were cultured from E18 Sprague–Dawley rat fetuses as previously described [(26), p. 28–40]. The bilateral cortices were dissected from 10 E18 rat embryo brains, the meninges were carefully removed, and the tissue was placed in a conical tube containing a mixture of Dulbecco's modified Eagle medium and Ham's F-12 medium (DMEM/F12 medium) on ice. The tissue was allowed to settle at the bottom of the tube, and the supernatant was then carefully removed to leave only the tissue covered by the medium. The tissue was enzymatically digested with 0.25% trypsin containing 0.5 mg/ml DNase for 20 min at 37°C, with gentle shaking of the tube every 5 min. Then, complete DMEM/F12 medium containing 10% fetal bovine serum (FBS) was added to the tube to terminate the digestion, and the mixture was passed through a cell strainer to obtain single cells and then centrifuged for 5 min at 1,000 rpm. The supernatant was removed, and the cells were re-suspended in complete DMEM/F12 medium containing 10% FBS, 1% penicillin/streptomycin, and 0.5 mmol/L l-glutamine by gentle pipetting. The cells were counted using trypan blue and a cell counter and then seeded into 24-well culture plates pre-coated with poly-l-lysine (0.1 g/L). Once the cells had attached ~4 h later, the plating medium was replaced with cell culture medium consisting of neurobasal medium, B27 (2%), bovine serum albumin (1%), and penicillin/streptomycin (1%). The cultures were maintained in an incubator at 37°C with 5% CO_2_.

### Assessment of the Purity of the Cultured Cells

Cell purity was assessed by immunostaining for neurofilament (NF), which is expressed in neurons, and glial fibrillary acidic protein (GFAP), which is expressed in astrocytes. After the primary neurons were cultured for 7 days, the cells were washed with cold phosphate-buffered saline (PBS) and fixed in 4% paraformaldehyde for 30 min at room temperature. Then, the cells were rinsed three times with PBS, blocked with 5% donkey serum for 1 h at room temperature, and then incubated with (1) mouse polyclonal anti-NF (1:200; Beijing Guan Xing Yu Science and Technology Co., Ltd., Beijing, China) and (2) rabbit polyclonal anti-GFAP (1:500; Beijing Guan Xing Yu Science and Technology Co., Ltd.) at 4°C overnight. After removal of the primary antibodies and rinsing, the cells were incubated with Alexa-Fluor 488-conjugated goat anti-mouse antibody (1:500; Invitrogen, Thermo Fisher Scientific, Inc.) and Alexa-Fluor 568-conjugated goat anti-rabbit antibody (1:500; Invitrogen, Thermo Fisher Scientific, Inc.) for 1 h at room temperature. The nuclei were stained with DAPI (4′,6-diamidino-2-phenylindole, 1:500, D1306; Invitrogen, CA, USA). An LSM-780 laser-scanning confocal microscope (Carl Zeiss Micro Imaging GmbH, Jena, Germany) was used to capture the images.

### Oxygen-Glucose Deprivation/Reperfusion Procedure

Confluent cultures grown for 7 days on 24-well culture plates were subjected to hypoxia (1% O_2_, 94% N_2_, and 5% CO_2_) in glucose-free medium for 2 h. The cultures were then re-oxygenated in neurobasal medium with 2% B-27 Serum-Free Supplement under normal conditions (5% CO_2_) (26).

### Assessment of Cultured Cell Viability

Cultured neuron cells exposed to oxygen-glucose deprivation/reperfusion (OGD/R) were treated with a range of DCS concentrations (0, 0.2, 0.4, 0.6, 0.8, or 1.0 μg/μl) for 4 h; cells were reoxygenated; and the blank control group (not exposed to OGD/R) was also included. Cell viability was detected used a cell counting kit (Cell Counting Kit-8; Dojindo, Kumamoto, Japan) in accordance with the manufacturer's instructions. Cell Counting Kit-8 solution was added to the medium at a dilution of 1:10, and the plate was incubated for 4 h. The absorbance at 450 nm was measured using SpectraMax M5 (Molecular Devices, Sunnyvale, CA, USA).

### Detection of Cultured Cell Apoptosis

Apoptosis was detected by using phycoerythrin (PE)–Annexin V Apoptosis Detection Kit I (BD559763; BD Biosciences, San Jose, CA, USA). Briefly, primary neuronal cells were washed twice with cold PBS, trypsinized, and re-suspended in 1× binding buffer at a concentration of 1 × 10^6^ cells/ml. A 100-μl volume of cell suspension (1 × 10^5^ cells) was transferred to a 5-ml FACS tube, and then 5 μl each of PE–Annexin V and 7-amino-actinomycin D (7-AAD) was added. The cells were gently vortexed and incubated for 15 min at 25°C in the dark. Then, 400 μl of 1× binding buffer was added to each tube, and the fluorescence was measured within 1 h at 520 nm (SpectraMax M5). For plotting of the results, PE–Annexin V was set as the horizontal axis and 7-AAD as the vertical axis: the upper-left quadrant indicated mechanically damaged cells, the upper-right quadrant apoptotic or necrotic cells, the lower-left quadrant normal cells, and the lower-right quadrant early apoptotic cells. The level of apoptosis was normalized to that observed in cells not exposed to OGD/R.

### TUNEL and DAPI Staining

In order to detect apoptosis, TUNEL staining was performed using the *in situ* Cell Death Detection kit for Fluorescein (Roche, China). After reoxygenation for 24 h, cortical neurons were stained with terminal deoxynucleotidyl transferase (TdT)-mediated dUTP nick end-labeling (TUNEL) according to the manufacturer's recommendations, and DAPI was used as counterstain. Negative controls were treated in the same way but were incubated without the TdT enzyme, while positive controls were treated with DNase I. In microscopic observation, TUNEL-positive showed green, and DAPI-positive showed blue. Six view fields were chosen randomly to calculate the percentage of apoptotic cells. The counts of positive stained cells from five observed fields were then averaged.

### Western Blot Experiments

Western blotting was performed as previously described with modifications [(27), p. e1025]. Cells were lysed in whole-cell lysis buffer (50 mM HEPES, 150 mM NaCl, 1 mM EGTA, 10 mM sodium pyrophosphate, 1.5 mM MgCl_2_, 100 mM NaF, 10% glycerol, and 1% Triton X-100, pH 7.2) containing an inhibitor cocktail (1 mM phenylmethylsulfonyl fluoride, 10 mg/ml aprotinin, and 1 mM sodium orthovanadate) to extract total protein. Protein concentrations were determined using a standard bicinchoninic acid assay (23225; Thermo Fisher Scientific Inc., Waltham, MA, USA), and 50 μg of total protein was subjected to 10% SDS-PAGE followed by electrotransfer onto nitrocellulose membranes. The membranes were blocked in 5% skimmed milk and then incubated overnight at 4°C with primary antibodies against rat BCL-2 (1:1,000; Cell Signaling Technology, Danvers, MA, USA), rat Bax (1:1,000; Cell Signaling Technology), or rat β-actin (1:20,000, TA-09; Zhongshan Golden Bridge Co., Ltd, Beijing, China). The membranes were then washed three times (10 min each) with Tris-buffered saline/Tween-20 (TBST) and incubated with horseradish peroxidase-conjugated secondary antibodies in 5% skimmed milk for 1 h at room temperature. After washing three times (10 min each) with TBST, immunoreactive signals were detected using enhanced chemiluminescence (Pierce, Rockford, IL, USA). Band intensity was quantified using ImageJ software (NIH). Relative protein expression was normalized to β-actin.

### Measurement of ROS Levels

For the detection of intracellular ROS level, we used ROS-sensitive probe 2′,7′-dichlorodihydrofluorescein diacetate (H2DCFDA, D-399; Invitrogen). H2DCFDA was dissolved in DMSO to obtain 10 mM stock solution and further diluted before use. Cells were incubated with 5 μM staining solution in PBS in the dark for 30 min at 37°C, then harvested with 0.05% trypsin–EDTA solution, suspended in fresh medium, and immediately analyzed with flow cytometer (Epics XL; Beckman Coulter, USA; 488 nm laser).

### Statistical Analysis

Statistical analyses were performed by Prism software. Data were presented as mean ± standard error of the mean (SEM). Statistical significance was evaluated by one-way analysis of variance (ANOVA) or Student's *t*-test. A two-tailed *p*-value of < 0.05 was considered statistically significant (^*^p < 0.05, ^**^p < 0.01, ^***^p < 0.0005, ^****^p < 0.0001).

## Results

### DCS Reduces Neurological Deficits and Infarct Size in Rats Subjected to Transient MCAO

**Figure 1:**
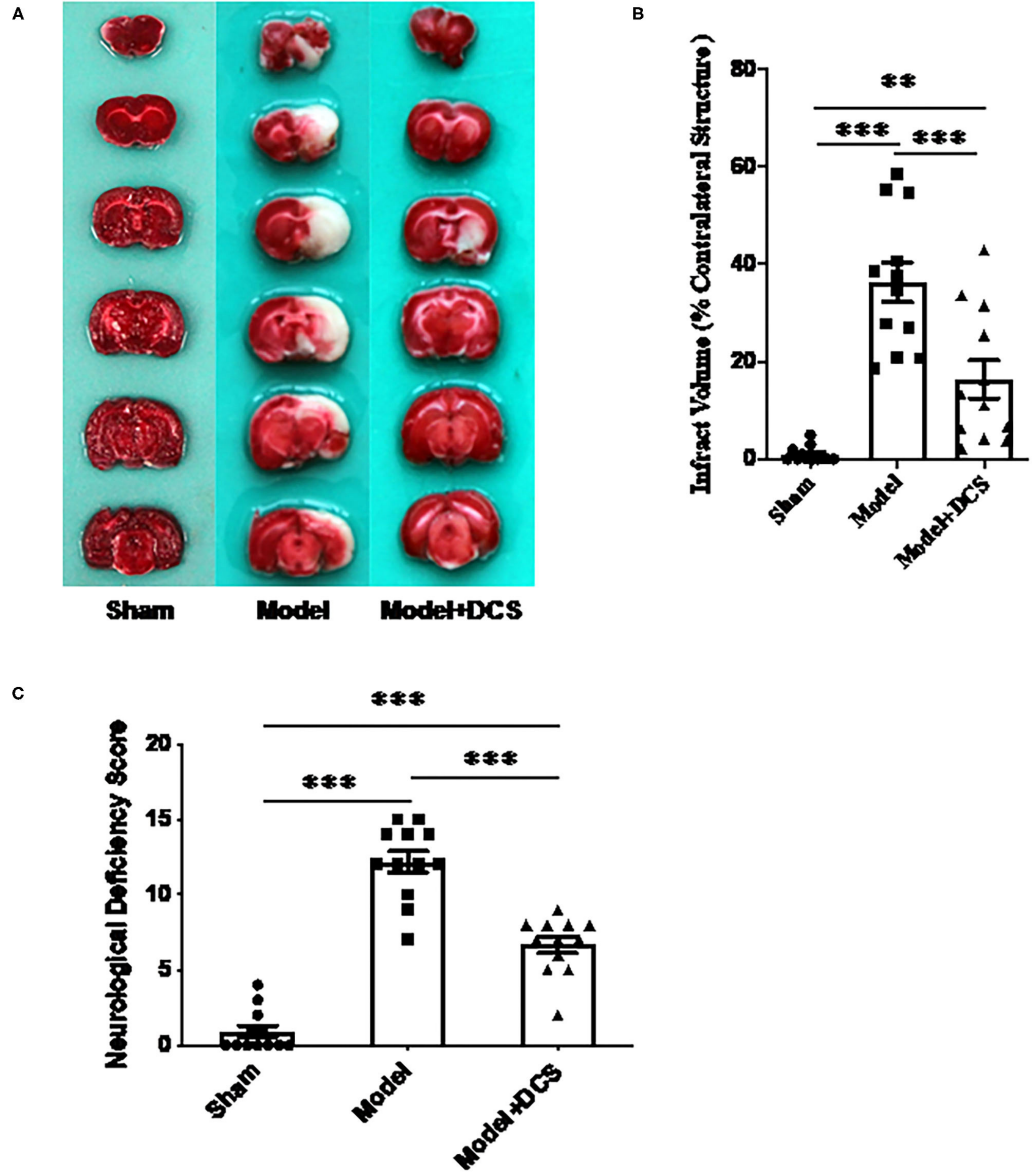
Effects of deproteinized calf serum (DCS) on neurological dysfunction and infarct volume in the rat model of ischemic stroke. **(A)** Representative photographs illustrating infarcted regions in the right side of the brain that had not stained with triphenyltetrazolium chloride (unstained areas were defined as infarcted tissue, while normal tissue was stained red). Sham: animals only went through anesthesia with isoflurane until CCA, ECA, lCA, and vagus nerve were exposed and isolated carefully then close the incision. Model + DCS: rats with ischemic stroke administered DCS in normal saline; Model: rats with ischemic stroke administered normal saline (vehicle) only. **(B)** Statistic analysis for **(A)**. Data shown as mean ± standard error of the mean (SEM). **p < 0.01, ***p < 0.0005. **(C)** Neurological dysfunction assessed using the neurological deficiency score (modified Bederson's method). Twelve rats per group were performed. Data shown as mean ± SEM. ***p < 0.0005.

In animals exposed to transient MCAO, infarcted tissue was found in the hemisphere ipsilateral to the occlusion (right side), and the infarcted region was consistent with the area supplied by the MCA (Figure 1A). Infarct volume percentage of contralateral brain volume in rats administered 0.125 mg/gbw DCS was 16.43 ± 4.64%, infarct volume percentage of rats treated with vehicle was 36.25 ± 4.63% compared with the vehicle group, and the infarct volume size was significantly reduced by DCS (^***^p < 0.005; Figure 1B).

In rats that had received transient MCAO, neurological deficit scores of animals treated with 0.125 mg/gbw DCS and vehicle were 6.67 ± 2.4 and 12.17 ± 1.9, respectively (^***^p < 0.0005; Figure 1C). The animal behavior test was rescued by DCS very much significantly.

### DCS Increases the Viability of Cultured Primary Neurons Exposed to OGD/R

**Figure 2:**
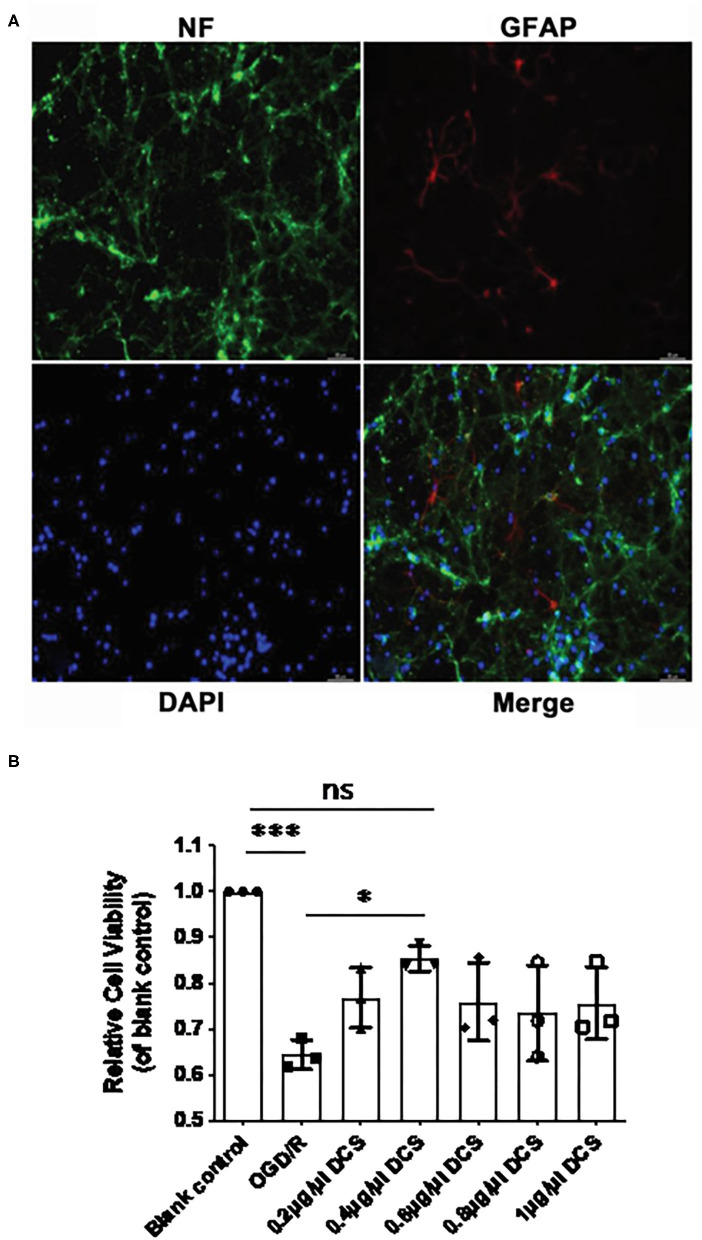
Effect of DCS on the viability of cultured primary neurons exposed to oxygen-glucose deprivation/reperfusion (OGD/R). **(A)** Cultured primary neurons isolated from rat E18 embryos and immunostained for neurofilament (NF, expressed in neurons; green) and glial fibrillary acidic protein (GFAP, expressed in astrocytes; red). Nuclei were stained with 4′,6-diamidino-2-phenylindole (DAPI; blue). Neuronal cell purity exceeded 95%. **(B)** Cell viability determined by using Cell Counting Kit-8. Primary neurons were exposed to OGD/R and administered DCS for 4 h at various concentrations (0, 0.2, 0.4, 0.6, 0.8, and 1 μg/μl). Cells not exposed to OGD/R were used as a blank control group. Data shown as mean ± SEM, *n* = 3. *p < 0.05, ***p < 0.0005.

According to our isolation protocol, after 7 days culture in conditional medium, the purity of the primary neurons was tested by immunofluorescence. Immunostaining for NF and GFAP revealed that the purity of the primary neuronal cells cultured from E18 embryo rats exceeded 95% (Figure 2A). The viability of cultured primary neurons unexposed to OGD/R was set as 100%, and the viability of primary neurons exposed to OGD/R was 64.4% to cells that were not exposed to OGD/R (^***^p < 0.0005; Figure 2B), which indicated that the OGD was successful. In cultured neurons subjected to OGD/R, cell viability in neurons treated with DCS at concentration of 0.2, 0.4, 0.6, 0.8, or 1.0 μg/μl was 76.7, 85.3, 75.9, 73.5, and 75.6%, respectively. Although all groups of DCS treatment showed improved cell viability compared with the OGD/R group, 0.4 μg/μl DCS had no significant changes compared with untreated cells (*p* > 0.05), but had significantly higher cell viability than cells not administered DCS (^*^p < 0.05; Figure 2B). Based on these results, subsequent experiments utilized a DCS concentration of 0.4 μg/μl.

### DCS Decreases the Apoptosis of Cultured Primary Neurons Exposed to OGD/R

**Figure 3:**
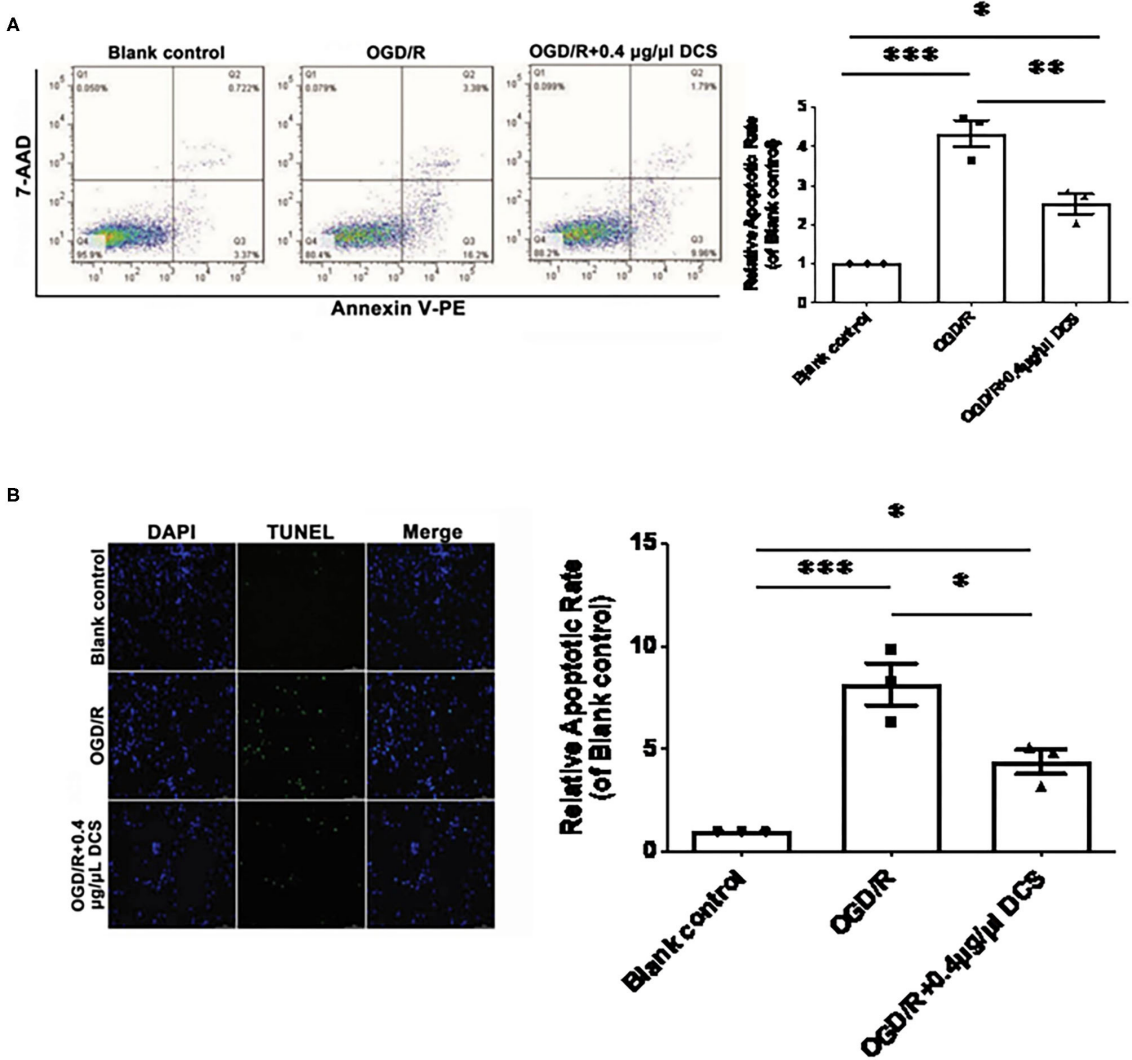
Effect of DCS on the apoptosis of cultured primary neurons exposed to OGD/R. **(A)** The apoptosis of cultured primary neurons isolated from rat E18 embryos assessed using a phycoerythrin/Annexin-V assay and flow cytometry. In the FACS picture, the upper-left quadrant represents mechanically damaged cells, the upper-right quadrant apoptotic or necrotic cells, the lower-left quadrant normal cells, and the lower-right quadrant early apoptotic cells. **(B)** The apoptosis of cultured primary neurons isolated from rat E18 embryos assessed using the terminal deoxynucleotidyl transferase dUTP nick end labeling (TUNEL) assay. The nuclei were stained with 4′,6-diamidino-2-phenylindole (DAPI; blue). Blank control: cells not exposed to OGD/R; OGD/R: cells exposed to OGD/R; OGD/R + 0.4 μg/μl DCS: cells exposed to OGD/R and treated with 0.4 μg/μl of DCS for 4 h. Data shown as mean ± SEM, *n* = 3. *p < 0.05, ***p < 0.005.

Primary neurons exposed to OGD/R exhibited a significantly higher level of apoptosis than cells not exposed to OGD/R, which was validated by 7AAD–PE–Annexin V staining and TUNEL staining. 7AAD–PE–Annexin V staining results showed that the apoptosis rate of OGD/R was 4.33 ± 0.34 times on untreated cells; however, the 0.4 μg/μl DCS group was 2.53 ± 0.34 times on untreated cells (Figure 3A). TUNEL staining results showed that the apoptosis rate of OGD/R was 8.13 ± 0.96 times on untreated cells, and the 0.4 μg/μl DCS group was 4.37 ± 0.96 times on untreated cells (Figure 3B).

Overall, 7AAD–Annexin V staining and TUNEL staining showed that the apoptosis level in cells exposed to OGD/R was rescued by the administration of 0.4 μg/μl DCS (^*^p < 0.05; ^**^p < 0.01; ^***^p < 0.0005; Figure 3).

### DCS Reduces the Intracellular Level of ROS in Cultured Primary Neurons Exposed to OGD/R

**Figure 4:**
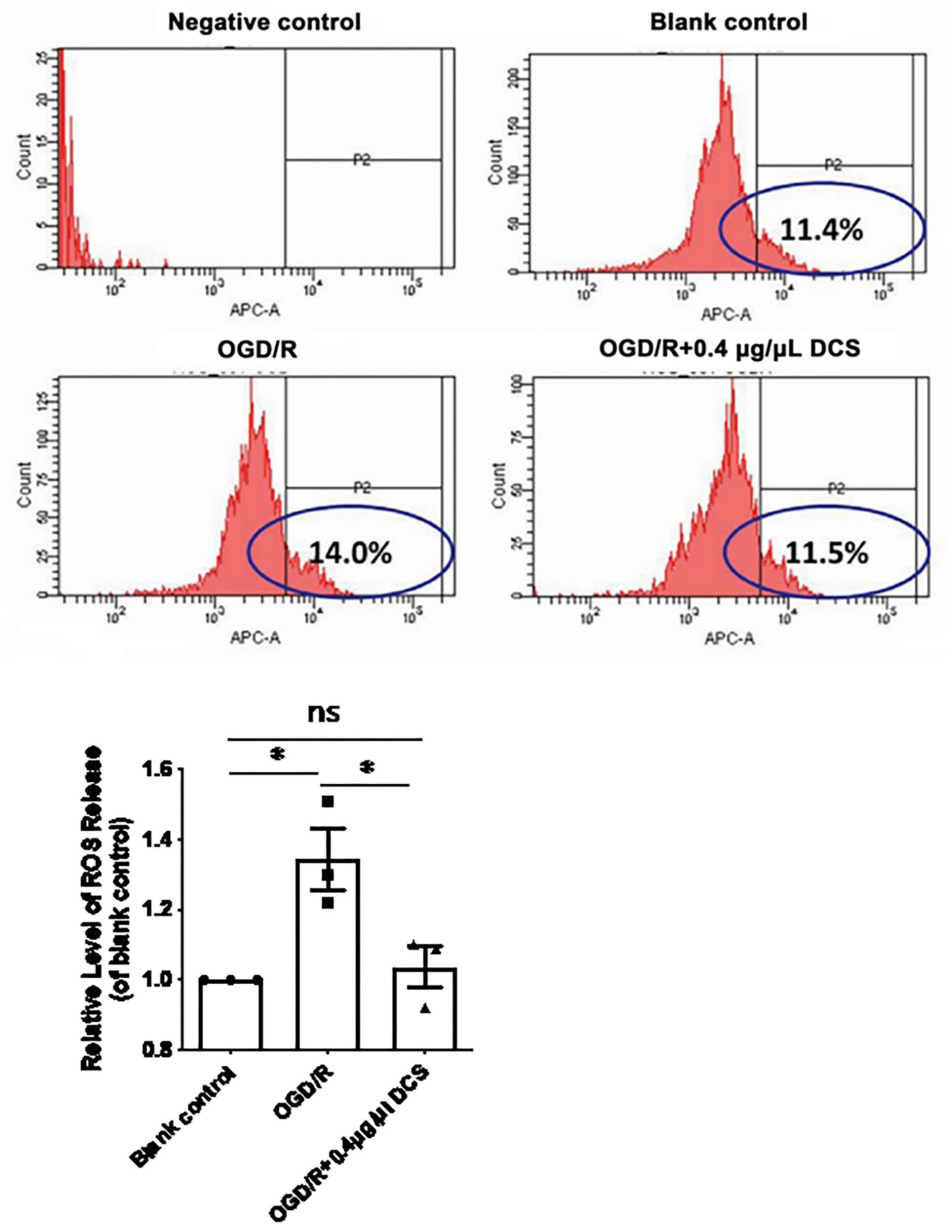
Effect of DCS on reactive oxygen species (ROS) levels in cultured primary neurons exposed to OGD/R. Flow cytometry analysis of ROS levels determined by using H2DCFDA staining. Blank control: cells not exposed to OGD/R; OGD/R: cells exposed to OGD/R; OGD/R + 0.4 μg/μl DCS: cells exposed to OGD/R and 0.4 μg/μl DCS for 4 h. Data shown as mean ± SEM, *n* = 3. *p < 0.05.

Relative level of intracellular ROS release caused by OGD/R increased from 1 to 1.34 ± 0.08 times higher in cultured neurons. Notably, treatment with 0.4 μg/μl DCS reduced the ROS release relative level to 1.04 ± 0.08 in cells subjected to OGD/R (^*^p < 0.05; Figure 4). DCS may protect the primary neuron *via* inhibiting the ROS release.

### DCS Downregulates Bax Protein Expression and Upregulates Bcl-2 Protein Expression in Cultured Primary Neurons Exposed to OGD/R

The Bcl2 family proteins are key regulators of apoptosis cell death. Bcl2-like subgroup, such as Bcl2, Bcl-xL, Mcl-1, Bcl-w, and A-1, suppresses cell death, and pro-apoptotic Bax-like subgroup, such as Bax, Bak, Bok, and Bik, promotes cell death. Bcl-2 and Bax were employed to check the role of DCS in cell apoptosis.

**Figure 5:**
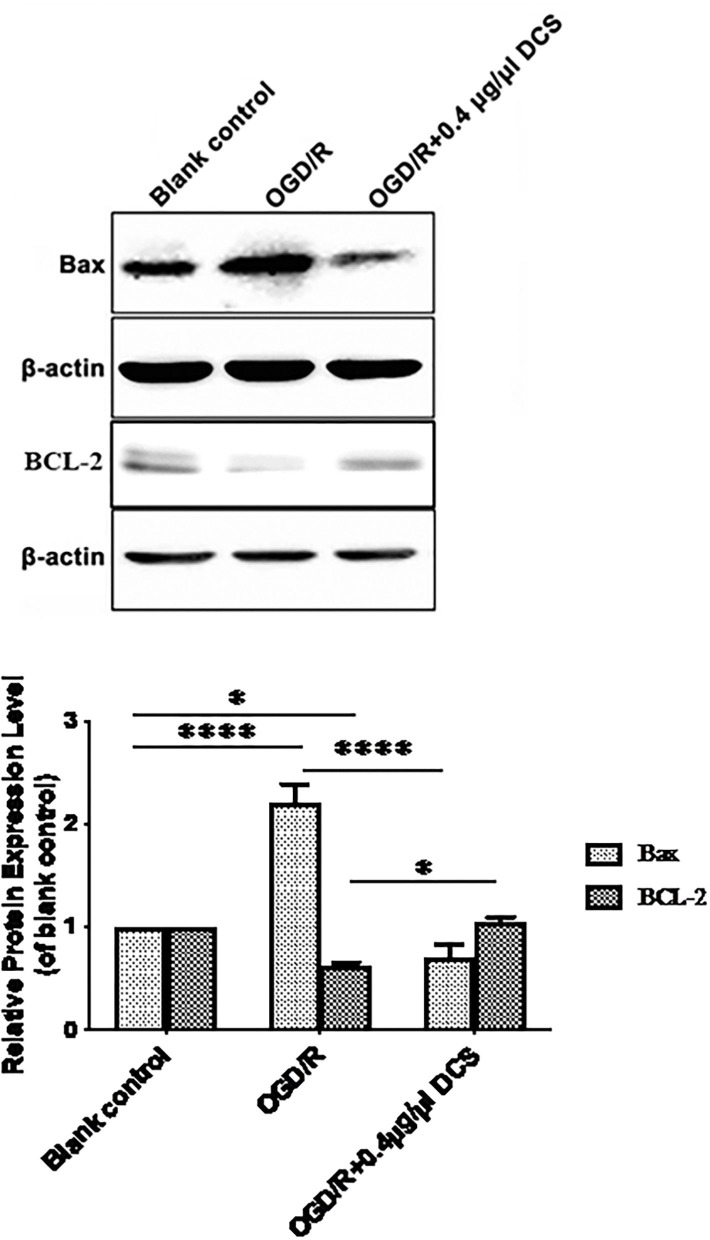
Effects of DCS on the expressions of Bax and Bcl-2 proteins in cultured primary neurons exposed to OGD/R. Expressions of Bax and Bcl-2 proteins were determined by Western blot. β-Actin was used as an internal control. Blank control: cells not exposed to OGD/R; OGD/R: cells exposed to OGD/R; OGD/R +0.4 μg/μl DCS: cells exposed to OGD/R and 0.4 μg/μl DCS for 4 h. Data shown as mean ± SEM, *n* = 3. *p < 0.05, ****p < 0.0001.

Primary neurons subjected to OGD/R showed 2.21 ± 0.13 times higher level of Bax protein expression and 0.62 ± 0.13 lower level of Bcl-2 protein expression than cells not exposed to OGD/R. In cells subjected to OGD/R, treatment with 0.4 μg/μl DCS downregulated the expression of Bax protein from 2.21- to 0.71-fold compared with the untreated cells. Expression of Bcl-2 protein was upregulated by DCS from 0.62- to 1.04-fold compared with untreated cell at the mean time (^*^p < 0.05; ^****^p < 0.0001; Figure 5).

## Discussion

Stroke is a major cause of morbidity and mortality in both developed and developing countries of the world. The only currently approved medical therapy for stroke is tPA, a thrombolytic agent that targets the thrombus within the blood vessel. A greater understanding of the pathophysiology of neuronal damage in ischemic stroke will help in the development of novel neuroprotective agents as additional management strategies. Important findings of our study were that 0.4 μg/μl DCS improved the neurological deficit and reduced infarct volume in rats subjected to MCAO. Furthermore, in primary neurons, 0.4 μg/μl DCS inhibited the effects of OGD/R to decrease cell viability, increase apoptosis, enhance intracellular ROS level, upregulate Bax, and downregulate Bcl-2. Taken together, our findings indicate that the neuroprotective effects of DCS following cerebral ischemia may, at least in part, be due to decreased ROS production and inhibition of apoptosis. Although not in this study, *in vivo* data have been verified by Zhou, Ziegler, and Guekht et al. as well [(16), p. 266–274, (28), p. 222–227, (29), p. 861–867].

Apoptosis is the main pathway through which oxidative stress leads to cell death, and neuronal apoptosis is a critical event underlying the mortality and morbidity associated with stroke. Although neural regeneration and plasticity can ameliorate, to some degree, the neurological deficits associated with ischemic stroke, these compensatory effects are limited. In agreement with previous studies in patients with ischemic stroke [(18), p. 873–875] and animal models [(19), p. 1623–1630], we found that DCS could reduce neurological deficits in a rat model of ischemic stroke. Furthermore, our observations indicated that DCS could reduce infarct volume in a rat model of ischemic stroke and protect neurons from apoptosis following OGD/R, which are consistent with other research in rats and primary neurons. These data suggest that the beneficial effects of DCS on neurological function following ischemic stroke are likely due to inhibition of the apoptosis pathway.

Important and novel findings of the present study were that DCS decreased the ROS levels induced by OGD/R in neuronal cells. These actions of DCS are likely to have contributed to the reduced level of apoptosis observed in neurons subjected to OGD/R. ROS is a collective term used to describe a number of species that contain one or more unpaired electrons, including superoxide, hydrogen peroxide, peroxyls, and hydroxyl radicals. Under normal conditions, ROS production is held in check primarily by antioxidant enzymes. However, when a cell becomes stressed, the production of ROS can exceed the antioxidant capacity and result in oxidative stress-related cell death. Mitochondrial respiratory chain complexes I and III are considered to be the major sites of cellular superoxide production [(30), p. 483–495, (31), p. 12–23, (32), p. 719–723], as ROS are produced as a byproduct of normal cellular metabolism of oxygen. The ROS produced during normal mitochondrial respiration are considered to be an important source of ROS for oxidative damage [(33), p. 62–70], and mitochondrial dysfunction is known to play a key role in various types of cell death [(34), p. 35–38, (35), p. 259–279, (36), p. 839–848]. Since glucose is the major energy substrate for cells of the CNS (astrocytes and neurons) under physiological conditions [(37), p. 33–41, (38), p. 281–300], DCS has been reported to enhance glucose uptake/oxidation, oxygen uptake/utilization, and cellular energy metabolism [(16), p. 266–274]. We suggest that these actions of DCS, at least in part, underlie its beneficial effects to reduce ROS levels and inhibit apoptosis in neuronal cells following an ischemic insult. However, further research is needed to elucidate the mechanisms involved.

## Conclusion

The neuroprotective effects of DCS following cerebral ischemia may in part be due to decreased ROS production and inhibition of apoptosis. Further studies are merited to clarify the mechanisms of DCS and establish whether DCS could be used as an effective treatment in patients with ischemic stroke.

## Data Availability Statement

The raw data supporting the conclusions of this article will be made available by the authors, without undue reservation.

## Ethics Statement

The animal study was reviewed and approved by The Ethics Committee of the Beijing Tiantan Hospital of Capital Medical University.

## Author Contributions

QW conceived and designed the experiments. WL and AG performed the experiments and wrote the paper. MS contributed reagents/materials analysis tools. JW analyzed the data. All authors have read and approved the final manuscript.

## Funding

This study was financially supported by the National Key R&D Program of China (Grant 2017YFC1307500), National Natural Science Foundation of China (81774053 and 81271285), Beijing–Tianjin–Hebei Cooperative Basic Research Program (H2018206435), and Science Technology and Innovation Committee of Shenzhen for Basic Research Projects (JCYJ20170307155449972).

## Conflict of Interest

The authors declare that the research was conducted in the absence of any commercial or financial relationships that could be construed as a potential conflict of interest.

## Publisher's Note

All claims expressed in this article are solely those of the authors and do not necessarily represent those of their affiliated organizations, or those of the publisher, the editors and the reviewers. Any product that may be evaluated in this article, or claim that may be made by its manufacturer, is not guaranteed or endorsed by the publisher.

## Abbreviations

DCS: deproteinized calf serum
MCAO: middle cerebral artery occlusion
OGD/R: oxygen-glucose deprivation/reperfusion
I/R: ischemia/reperfusion
ROS: reactive oxygen species
TTC: triphenyltetrazolium chloride
tPA: tissue-type plasminogen activator
CCA: common carotid artery
ECA: external carotid artery
ICA: internal carotid artery
NF: neurofilament
GFAP: glial fibrillary acidic protein
TUNEL: terminal deoxynucleotidyl transferase (TdT)-mediated dUTP nick end-labeling.
